# Lung Damage in Rheumatoid Arthritis—A Retrospective Study

**DOI:** 10.3390/ijms24010028

**Published:** 2022-12-20

**Authors:** Georgiana Dinache, Claudiu Costinel Popescu, Corina Mogoșan, Luminita Enache, Mihaela Agache, Cătălin Codreanu

**Affiliations:** 1 Rheumatology Department, “Carol Davila” University of Medicine and Farmacy, 020021 Bucharest, Romania; 2 “Dr. Ion Stoia” Clinical Center for Rheumatic Diseases, 020983 Bucharest, Romania

**Keywords:** interstitial pulmonary fibrosis, pulmonary nodules, rheumatoid arthritis

## Abstract

The current study aimed to evaluate rheumatoid arthritis (RA) patients with interstitial lung disease (ILD) in clinical practice and whether disease characteristics are associated with X-ray and high-resolution computed tomography (HR-CT) findings. Medical history of RA patients from a tertiary rheumatology clinic was retrieved from its electronic database starting from 1 January 2019 until the study date (8 August 2022) using International Classification of Disease version 10 codes for RA, ILD and exclusion criteria. The study included 78 RA patients (75.6% women, 15.4% active smokers), with average time from RA to ILD of 5.6 years. Regarding chest X-ray findings, men had a higher prevalence of nodules, combined fibrosis and nodules and combined bronchiectasis and nodules, rheumatoid factor (RF)-positive patients had a higher prevalence of fibrosis and anti-cyclic citrullinated peptide antibodies (ACPA)-positive patients had a higher prevalence of bronchiectasis. Regarding HR-CT findings, patients actively treated with methotrexate had a higher prevalence of nodules; a combination of fibrosis and nodules; combination of emphysema and nodules; and combination of fibrosis, emphysema and nodules. ILD develops within approximately 5 years from RA diagnosis, and ILD-associated imaging findings on chest X-rays and HR-CT are more prevalent among men with RA, among patients with positive RA serology (RF and/or ACPA) and RA patients on methotrexate.

## 1. Introduction

Rheumatoid arthritis (RA) is a systemic, progressive autoimmune disease characterized by joint and extraarticular manifestations. The lung is a common extraarticular target. Pulmonary manifestations in RA may include airway, parenchyma, pleura or vessel involvement. Pulmonary manifestations in RA usually occur after joint manifestations, but in certain circumstances lung damage can be the first manifestation of RA and in this situation, it is one of the most aggressive forms [1]. Thus, rheumatologists must be aware of these manifestations in patients with RA. The 2013 classification of the American Thoracic Society and the European Respiratory Society of idiopathic interstitial pneumonitis highlights the latest histopathological classifications of interstitial lung disease (ILD), many of which are also found in association with RA [2].

The pathophysiological changes underlying the development of ILD in patients with RA still remain insufficiently known. Data available in studies to date suggest the involvement of environmental factors and genetic factors. The presence of HLA-B54, HLA-DQB1*0601, HLA-B40 and HLA-DR4 has been associated with RA-ILD [3,4,5,6]. There are some studies that hypothesize that the lung is the main site of immunological disorder from which the development of RA starts [7]. Citrullinated proteins have been identified in bronchoalveolar lavage fluid in patients smoking without RA, and RA-specific antibodies have been detected in the sputum of patients at risk of developing RA [8,9].

It is hoped that future studies will be able to identify biomarkers or key molecules of RA-ILD, which will provide more information on the immunopathogenesis of the disease, so that a diagnosis can be made as early as possible. Up to this point, several RA-ILD-associated peptides have been identified, including anti-citrullinated peptide antibodies (ACPA), anti-vimentine antibodies, citrulline isoforms of heat shock protein 90, matrix metalloproteinase 1 (MMP) and inducible protein interferon-gamma 10 [10,11,12]. The exact role of these proteins is not fully known in terms of RA manifestations.

ILD is a heterogeneous group of diseases that affects the lung parenchyma through inflammatory and/or fibrosing mechanisms. ILD can be associated with environmental exposure (drugs, radiation, occupations and infections, including COVID-19 [13]) or with autoimmune diseases, especially systemic sclerosis and RA, which can develop into progressive fibrosing phenotype [14]. While in RA, the major advances made are early diagnosis, early aggressive treatment and modern therapeutic molecules; RA-associated lung damage is not yet sufficiently studied and regulated in terms of diagnosis, monitoring and therapeutic management. Interstitial pulmonary fibrosis (IPF) is associated with increased mortality, which is why early diagnosis and treatment are essential. Genetic susceptibility, smoking, male sex, rheumatoid factor (RF) and ACPA positivity are risk factors for IPF in RA. It is recommended that the diagnosis, clinical examination, functional testing, imaging and treatment strategy of ILD to be made through standardized management, within a multi-disciplinary team, which must include a pulmonologist, radiologist and rheumatologist [15,16].

Clinically, most frequently the patient is asymptomatic, but in the advanced stages exercise dyspnea and dry cough appear on a background of underlying rheumatic disease manifestations, and the patient reveals at examination bilateral adventitious basal crackle sounds. The gold standard of lung function evaluation is the combination of spirometry/plethysmography (especially for diffusing capacity for carbon monoxide—DLCO [17,18,19], and forced vital capacity—FVC), arterial blood gas analysis [20,21,22] and a stress test (e.g., the 6 min walking test—6MWT [18,23]). Standard anterior chest X-rays study is the first imaging investigation to be performed in a patient suspected of lung damage. Although it cannot offer a positive diagnosis, it can guide the differential diagnosis and it can suggest the severity of the disease [24].

The positive diagnosis of ILD is based entirely on high-resolution computed tomography (HR-CT), which provides information about the anatomy (e.g., “ground-glass” cross-linked opacities or “honeycomb” lesions), imaging pattern (e.g., usual interstitial pneumonia—UIP, characterized by peripheral subpleural and basal reticulations, with “honeycomb” lesions and traction bronchiectasis, or non-specific interstitial pneumonia—NSIP, characterized by “ground-glass” opacities, condensations and reticulations in the basal areas, which may be accompanied by traction bronchiectasis) [24,25,26,27,28,29], evolution over time, clues related to the underlying disease [30] and prognosis of the disease. The progressive character of ILD has been defined if the progression occurs despite the current optimal management and treatment, which includes glucocorticoids and immunosuppressive therapy, at which point the need of antifibrotic treatment appears [18,31]. Unfortunately, clinical practice reveals that extraarticular manifestations of RA, especially ILD, are frequent and underdiagnosed. Thus, the current study aimed to evaluate RA patients with lung damage in clinical practice and whether disease characteristics are associated with X-ray and HR-CT findings.

## 2. Results

### 2.1. Demographics

The study included 78 RA patients, of whom 75.6% were women, with an average RA duration of 11.8 years and an average duration of ILD of 4 years. The average age at RA diagnosis was 56.5 years, the average age at ILD diagnosis was 64.2 years and the average age at the introduction into the study was 68.2 years (Table 1). Of note, the mean time elapsed from RA diagnosis to ILD diagnosis was 5.6 years. Surprisingly, only 12 (15.4%) patients were active smokers, and 7 (9.0%) patients were former smokers, while the rest of the patients were not significantly exposed to smoking.

### 2.2. RA Characteristics

Regarding RA serology, 51 (65.4%) patients had positive RF, 52 (66.7%) patients had positive ACPA and 43 (55.1%) patients had both RF- and ACPA-positive titers. Regarding current treatment, 87.2% of patients were on active csDMARD treatment, 71.8% on one csDMARD molecule and 15.4% on a combination of two csDMARDs. The most frequent csDMARD was leflunomide (42.3%), followed by both methotrexate (19.2%) and hydroxychloroquine (19.2%; Table 2). Additionally, 20.5% of patients were on bDMARDs, either in monotherapy (25.0%) or combined with a csDMARD (75.0%). The most frequent bDMARDs were etanercept (6.4%), adalimumab (5.1%) and rituximab (3.8%, Table 2). Patients’ history revealed that 42 patients (53.8%) had previous exposure to methotrexate, 23 (29.5%) patients to leflunomide, 25 (32.1%) patients to sulfasalazine, 11 (14.1%) patients to hydroxychloroquine, 8 (10.3%) patients to azathioprine and 3 (3.8%) patients to cyclosporine.

### 2.3. Chest X-ray Findings

The data suggest a difference in chest X-ray findings depending on sex: compared to women, men had a higher prevalence of nodules (42.1% versus 20.3%, *p* = 0.049), which also explained their higher prevalence of combined fibrosis and nodules (36.8% versus 13.6%, *p* = 0.025) and their higher prevalence of combined bronchiectasis and nodules (36.8% versus 13.6%, *p* = 0.025; Table 3). Compared to RA patients without fibrosis on chest X-rays, RA patients with fibrosis had a significantly higher median age at ILD diagnosis, namely 66 (31–81) years compared to 58 (37–82) years (*p* = 0.047).

**Figure 1:**
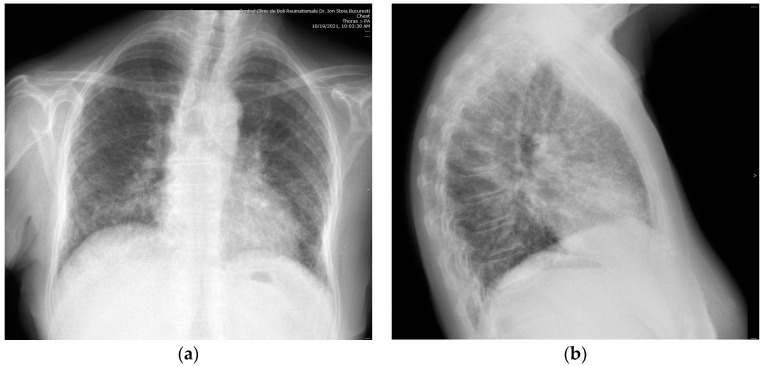
Radiographic ILD in a 71-year-old woman with seropositive RA showing diffuse interstitial fibrosis with bilateral reticular and areolar pattern (“honeycomb”) on (**a**) anteroposterior view and on (**b**) left lateral view.

RA serology was associated with worse lung findings on chest X-rays, a hypothesis suggested by the following four observations: compared to seronegative RA patients, patients with positive RF had a higher prevalence of fibrosis (78.4% compared to 55.6%, *p* = 0.035; Figure 1); compared to patients with negative ACPA, patients with positive ACPA had a higher prevalence of bronchiectasis (19.2% compared to 3.8%, *p* = 0.046); compared to patients negative for both RF and/or ACPA, patients with positive RA and ACPA had a higher prevalence of bronchiectasis (20.9% compared to 5.7%, *p* = 0.045) and a higher prevalence of combined fibrosis and bronchiectasis (11.6% compared to 0.0%, *p* = 0.037).

RA treatment molecules seem to have an impact on chest X-ray findings. Compared to patients without the specific csDMARD or combination of csDMARDs, patients on cyclosporine and patients on mycophenolate mofetil had a respective higher prevalence of combined fibrosis and bronchiectasis (100% compared to 5.2%, *p* = 0.044 for both). Similarly, patients on combined methotrexate and leflunomide had a higher prevalence of combined fibrosis and emphysema (100% compared to 2.6%, *p* = 0.038), and patients on combined leflunomide and sulfasalazine had a higher prevalence of emphysema (100% compared to 14.5%, *p* = 0.026) and of combined emphysema and bronchiectasis (50.0% compared to 0.0%, *p* = 0.026). The lack of csDMARDs seems to be associated with X-ray findings, since, compared to patients on combined therapy (csDMARD and bDMARD), patients on bDMARD monotherapy had a higher prevalence of nodules (46.7% compared to 20.6%, *p* = 0.038).

### 2.4. HR-CT Findings

**Figure 2:**
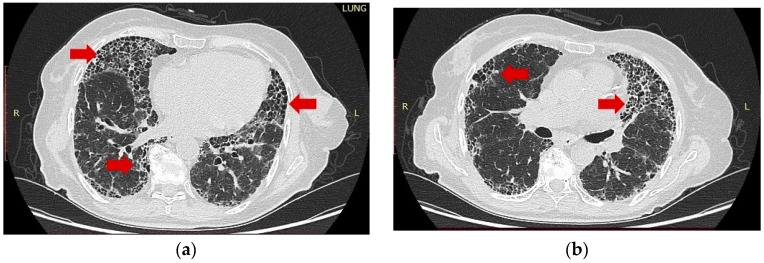
HR-CT scan of the same patient (cross-sections) showing bilateral moderately severe “honeycomb” fibrosis (red arrows) in an “usual interstitial pneumonia” pattern: (**a**) predominantly peripheral interstitial changes; (**b**) basal subpleural disposition of fibrosis thickened septa.

A subgroup of 33 patients (42.3%) underwent lung HR-CT (Table 3; Figure 2). This subgroup of patients, although small, revealed significant associations. As in the case of chest X-ray findings, compared to patients with negative ACPA, patients with positive ACPA had a higher prevalence of nodules on CT (56.0% versus 12.5%, *p* = 0.046). Similarly, compared to patients without current methotrexate, patients actively treated with methotrexate had a higher prevalence of nodules (100% compared to 35.7%, *p* = 0.013); combination of fibrosis and nodules (80.0% compared to 32.1%, *p* = 0.046); combination of emphysema and nodules (40.0% compared to 3.6%, *p* = 0.043); and combination of fibrosis, emphysema and nodules (40.0% compared to 3.6%, *p* = 0.043). Compared to patients on combined therapy (csDMARD and bDMARD), patients on bDMARD monotherapy had a higher prevalence of combined fibrosis and bronchiectasis (70.0% compared to 26.1%, *p* = 0.026); combined bronchiectasis and nodules (50.0% compared to 13.0%, *p* = 0.036); and combined fibrosis, bronchiectasis and nodules (50.0% compared to 8.7%, *p* = 0.016).

### 2.5. Lung Function Tests

A subgroup of 15 patients (19.2%) had lung function tests results, which were normal in two cases (13.3% of subgroup) or revealed a pattern of obstructive lung disease in three cases (20.0%), restrictive lung disease in nine cases (60.0%) and low DLCO in nine cases (60.0%).

## 3. Discussion

ILD is one of the most common extraarticular manifestations of RA and has been observed in up to 58% of patients. ILD is more common in men, as our study has shown: the results confirm that men had a higher prevalence of pulmonary nodules compared to women, which also explains the higher prevalence of pulmonary fibrosis associated with the pulmonary nodules. Additionally, risk factors, such as smoking, RF and ACPA positive serology, increase the prevalence of lung damage in patients with RA. In the current study, 55.1% of patients had both RF-positive and ACPA-positive results. Positive serology has been associated with a higher prevalence of pulmonary fibrosis compared to patients with negative ACPA. Surprisingly, out of the 78 patients, only 19 were exposed to smoking before the study, 12 being active smokers and 7 being ex-smokers. Even so, pulmonary nodules and functional impairment are known to be prevalent among ex-smokers [32].

From the point of view of medication, patients were exposed to the most available csDMARDs, which vary primarily depending on the evolution of RA disease activity and progression. Most of the time, there is a tendency of both rheumatologists and pulmonologists to interrupt treatment with methotrexate when lung damage occurs in the evolution of the disease. Thus, while the interruption of methotrexate is correct in the case of rheumatoid lung nodules, in the case of ILD in RA, there is still not enough evidence to support its association with methotrexate exposure. Thus, in RA-associated ILD, it is very important to collaborate closely between the specialists who diagnose, monitor and treat the patient, increasing the importance of achieving a multidisciplinary team consisting of the rheumatologist specialist, the pulmonologist specialist and the imaging specialist, a group that will act according to a well-established consensus. This will implicitly lead to an accurate diagnosis, as early as possible and to adequate and individualized treatment. Continuing this research, a prospective study will be conducted that will include patients with early and established RA and with normal chest X-rays who will be followed (questionnaire, clinical examination, imaging) to identify incident cases of ILD and its risk factors in RA, ultimately aiming to treat them as early as possible in order to increase the quality of life and to decrease morbidity and mortality.

RA is the most chronic inflammatory rheumatic disease, with a prevalence of 0.5–2% in the general population [33]. The disease is more common in women than in men, with a sex ratio of 3:1. Extraarticular manifestations occur in about 50% of patients, and the lung is among the most frequent sites [34]. Pulmonary manifestations occur in approximately 67% of patients with RA, although some studies indicate a lower incidence (10–20%) [35,36,37]. This large variation reflects the differences in the design of the studies, the differences in the study population and how pulmonary involvement is defined in RA. Many of the patients with RA do not show clinical manifestations of lung damage despite their diagnosis on imaging or functional samples, which leads to underdiagnosis. In a study with 52 patients with RA, HR-CT identified pulmonary manifestations in 67.3% of patients, and 40% of them had respiratory symptoms [35]. In addition to ILD, drug toxicity and secondary lung infections are sources of pulmonary manifestations to be taken into account in patients with RA. Mortality in patients with RA with extraarticular manifestations is higher compared to those without extraarticular manifestations. Mortality in RA is higher in the first 5–7 years after diagnosis, with a higher risk in men than in women with a mortality ratio of 2.07:1.97 [38,39]. Lung disease has a mortality of 10–20% in patients with RA, most of which is attributed to ILD [40,41,42].

HR-CT significantly increases the sensitivity and accuracy of RA-ILD diagnosis compared to standard radiography. In a study with 150 patients with RA, HR-CT revealed in 19% of patients the presence of ILD. In the same study, in less than 3% of patients, standard X-rays revealed bilateral interstitial infiltrate [36]. The most common form found on standard radiography was the UIP pattern, which is characterized on HR-CT by predominantly basal bilateral interstitial reticular infiltrate, “honeycomb” and traction bronchiectasis. Respiratory symptoms, especially dyspnea, can be difficult to assess in patients with significant limitation of joint mobility. Thus, doctors must remain alert to subtle symptoms, which may include recently installed dry cough, mild-to-moderate physical asthenia and decreased oxygen saturation [36,43]. Pulmonary biopsy is usually not indicated in patients with RA-ILD. However, if the diagnosis is tentative or the changes on HR-CT are atypical, the pulmonary biopsy may be useful.

## 4. Materials and Methods

### 4.1. Patients

Medical history of RA patients from a tertiary rheumatology clinic was retrieved from its electronic database starting from 1 January 2019 until the study date (8 August 2022; Table 4). International Classification of Disease version 10 codes were used to identify adult in-patients or out-patients with RA whose diagnosis also fulfilled the current classification criteria [44], without concomitant connective tissue diseases (overlap syndromes), but with lung involvement proven by at least one imaging method (standard chest X-ray, HR-CT). All patients gave written informed consent for medical management and scientific use of data at the time of evaluation in the clinic.

### 4.2. Variables

Electronic data records revealed demographic data (sex, date of birth), smoking status, RA history (date of diagnosis), phenotype (RF and ACPA status, both determined by the local laboratory using commercially available enzyme-linked immunosorbent assay kits) and treatment principles (disease-modifying antirheumatic drugs—DMARDs, either conventional synthetic—csDMARDs, biologic—bDMARDs or targeted synthetic—tsDMARDs) as well as information regarding RA-associated ILD (date of diagnosis, standard chest X-ray, HR-CT and lung function tests—spirometry/plethysmography). Smoking exposure included patients who were active smokers and ex-smokers (patients who quit smoking within the last decade prior to study inclusion). The acquired dates allowed us to calculate age at study inclusion (difference in years between birth date and study inclusion date), age at RA diagnosis (difference in years between birth date and RA diagnosis date), RA duration (difference in years between RA diagnosis date and study inclusion date), age at ILD diagnosis (difference in years between birth date and ILD diagnosis date), ILD duration (difference in years between ILD diagnosis date and study inclusion date) and time from RA to ILD (difference in years between ILD diagnosis date and RA diagnosis date). All chest X-rays were performed and interpreted in the clinic by the same imaging specialist, while HR-CT scans were performed both through the clinic and in collaboration with pulmonology specialists who follow the patients for lung involvement. Spirometry testing followed the American Thoracic Society (ATS)/European Respiratory Society (ERS) recommendations [45].

### 4.3. Statistics

Data distribution normality was assessed using descriptive statistics, normality, stem-and-leaf plots and the Lillefors corrected Kolmogorov–Smirnov tests. Continuous variables are reported as “mean ± standard deviation” if normally distributed, or as “median (minimum-maximum)” if non-normally distributed, while dichotomous variables are reported as “percentage of group or subgroup”. Mann–Whitney U tests were used to assess differences in continuous variables among subgroups of dichotomous categorical variables, while the associations between categorical variables were studied using χ^2^ or Fisher’s exact tests. The statistical tests were considered significant if *p* < 0.05. The statistical analysis was performed using IBM SPSS Statistics version 25.0 for Windows (IBM Corp., Armonk, NY, USA).

## 5. Conclusions

The cross-sectional observation of RA patients revealed that ILD develops within approximately five years from RA diagnosis and that ILD-associated imaging findings on chest X-rays and HR-CT are more prevalent among men with RA, among patients with positive RA serology (RF and/or ACPA) and RA patients on methotrexate. Surprisingly, only 24.4% of patients were active smokers and former smokers, while the rest of the patients had not been significantly exposed to smoking. The data suggest a difference in chest X-ray findings depending on sex: compared to women; men had a higher prevalence of nodules, which also explained their higher prevalence of combined fibrosis and nodules and their higher prevalence of combined bronchiectasis and nodules. Compared to RA patients without fibrosis on chest X-rays, RA patients with fibrosis had a significantly higher median age at ILD diagnosis. RA serology was associated with worse lung findings on chest X-rays: compared to seronegative RA patients, patients with positive RF had a higher prevalence of fibrosis; compared to patients with negative ACPA, patients with positive ACPA had a higher prevalence of bronchiectasis; and compared to patients negative for both RF and/or ACPA, patients with positive RA and ACPA had a higher prevalence of bronchiectasis and a higher prevalence of combined fibrosis and bronchiectasis. Prospective studies are needed to confirm these risk factors and to standardize diagnostic and management principles in RA-associated ILD.

## Figures and Tables

**Table 1:** Demographics of RA patients (n = 78).

Women	75.6%
active smokers	15.4%
ex-smokers	9.0%
smoking exposure (%)	24.4%
smoking exposure (pack-years)	33 (7)
age at study inclusion (years)	68.2 (9.8)
age at RA diagnosis (years)	56.5 (12.4)
RA duration (years)	11.8 (8.4)
age at ILD diagnosis (years)	64.2 (10.3)
ILD duration (years)	4.0 (1.8)
time from RA to ILD (years)	5.6 (0–42)

Abbreviations: ILD—interstitial lung disease; RA—rheumatoid arthritis; SD—standard deviations.

**Table 2:** Current DMARD therapy of RA patients (n = 78).

MTX	19.2%	HCQ + AZA	1.3%
LEF	42.3%	HCQ + CsA	1.3%
SSZ	10.3%	LEF + HCQ	3.8%
HCQ	19.2%	abatacept	2.6%
AZA	9.0%	adalimumab	5.1%
CsA	1.3%	baricitinib	2.6%
MMF	1.3%	etanercept	6.4%
MTX + LEF	1.3%	nintedanib	1.3%
MTX + SSZ	1.3%	rituximab	3.8%
MTX + HCQ	2.6%	tocilizumab	2.6%
LEF + SSZ	2.6%	tofacitinib	1.3%

Non-reported combinations of two and three csDMARDs returned zero results; Abbreviations: AZA—azathioprine; b/cs/tsDMARDs—biological, conventional synthetic or targeted synthetic disease-modifying antirheumatic drugs; CsA—cyclosporine; LEF—leflunomide; HCQ—hydroxychloroquine; MMF—mycophenolate mofetil; MTX—methotrexate; RA—rheumatoid arthritis; SSZ—sulfasalazine.

**Table 3:** Chest X-ray and HR-CT findings in RA patients (n = 78).

Chest X-ray		HRCT	
normal	6.4%	normal	0
ILD	70.5%	ILD	84.8%
BE	14.1%	BE	45.5%
E	16.7%	E	21.2%
N	25.6%	N	45.5%
ILD + BE	6.4%	ILD + BE	39.4%
ILD + E	3.8%	ILD + E	18.2%
ILD + N	19.2%	ILD + N	39.4%
BE + E	1.3%	BE + E	18.2%
BE + N	1.3%	BE + N	24.2%
E + N	1.3%	E + N	9.1%
		ILD + BE + E	15.2%
		ILD + BE + N	21.2%
		BE + E + N	6.1%
		ILD + E + N	9.1%
		ILD + BE + E + N	6.1%

Other non-reported combinations of three or four different pathologic findings returned zero results. Abbreviations: BE—bronchiectasis; E—emphysema; HR-CT—high-resolution computed tomography; ILD—interstitial lung disease (fibrosis on X-rays and “honeycomb” or “ground-glass” opacities on HR-CT); N—(micro)-nodules; RA—rheumatoid arthritis.

**Table 4:** Flow chart of inclusion of patients (n = 78).

	←	ICD codes for RA within timeframe
n = 3090		
↓	←	ICD codes for ILD
n = 321		
↓	←	ICD codes for overlapping CTD
n = 307		
↓	←	lung CT procedure and data
n = 84		
↓	←	RA classification criteria
n = 78		

Abbreviations: CT—computer tomography; CTD—connective tissue disease; ICD—International Classification of Diseases; ILD—interstitial lung disease; RA—rheumatoid arthritis.

## Data Availability

The data presented in this study are available on request from the corresponding author. The data are not publicly available due to patient confidentiality.
